# Short-Term Outcomes and Stoma-Related Morbidity of Diverting Ileostomy After Laparoscopic Total Mesorectal Excision Following Neoadjuvant Chemoradiotherapy for Mid- and Distal Rectal Cancer: A Retrospective Single-Center Study

**DOI:** 10.3390/jcm15197418

**Published:** 2026-09-24

**Authors:** Murat Derebey, Ufuk Karabacak, Ismail Alper Tarim, Enes Yasin Albayrak, Selcuk Gurz, Saim Savas Yuruker

**Affiliations:** 1 Department of General Surgery, Ondokuz Mayıs University Faculty of Medicine, 55270 Samsun, Turkey; 2 Department of General Surgery, Bingol State Hospital, 12000 Bingol, Turkey; 3 Department of Thoracic Surgery, Ondokuz Mayıs University Faculty of Medicine, 55270 Samsun, Turkey

**Keywords:** rectal cancer, neoadjuvant chemoradiotherapy, total mesorectal excision, diverting ileostomy, stoma-related morbidity

## Abstract

**Background and Objectives:** Diverting ileostomy (DI) is frequently used after low rectal cancer surgery to mitigate the clinical consequences of anastomotic leakage (AL). However, its potential benefits must be balanced against stoma-related morbidity. This study aimed to evaluate short-term postoperative outcomes and stoma-related morbidity associated with DI after laparoscopic total mesorectal excision (TME) following neoadjuvant chemoradiotherapy (nCRT) for mid- and distal rectal cancer. **Methods:** This retrospective single-center cohort study included 104 of 233 patients assessed for eligibility who had locally advanced mid- or distal rectal adenocarcinoma and underwent TME using an initial laparoscopic approach following nCRT between 2020 and 2025. Patients were stratified according to DI status. Preoperative, operative, pathological, and postoperative outcomes, including AL, 30-day postoperative complications, readmission, surgical reintervention, and stoma-related morbidity, were compared. Propensity score overlap weighting was used as the primary adjusted analysis to address confounding by indication. **Results:** A total of 50 patients (48.1%) underwent DI and 54 (51.9%) did not. Patients in the DI group had more distal tumors and more frequently underwent ultra-low anterior resection or intersphincteric resection. After propensity score overlap weighting, DI was not significantly associated with non-stoma-related complications within 30 days (adjusted RR, 0.935; 95% CI, 0.566–1.543; *p* = 0.792) or overall 30-day morbidity (adjusted RR, 1.301; 95% CI, 0.893–1.895; *p* = 0.158). However, DI was associated with major morbidity (adjusted RR, 4.658; 95% CI, 1.477–14.688; *p* = 0.004) and 30-day readmission (adjusted RR, 3.665; 95% CI, 1.397–9.621; *p* = 0.003). Nine patients in the DI group (18.0%) required reoperation within 30 days for stoma-related complications. **Conclusions:** After adjustment for baseline differences, DI was not significantly associated with overall or non-stoma-related 30-day morbidity, whereas associations with major morbidity and 30-day readmission persisted. Serious stoma-related complications may represent an important component of the early postoperative treatment burden associated with DI. These findings support careful patient selection when balancing the potential benefits of fecal diversion against stoma-related morbidity.

## 1. Introduction

Total mesorectal excision (TME) following neoadjuvant chemoradiotherapy (nCRT) remains the cornerstone of curative treatment for patients with locally advanced rectal cancer [1]. The widespread adoption of minimally invasive techniques has improved perioperative recovery while maintaining oncological outcomes comparable to those of open surgery [2,3]. Despite these advances, anastomotic leakage (AL) remains one of the most serious postoperative complications following rectal cancer surgery. The reported leakage rates vary widely, ranging from 1.2% to 27%, and their development adversely affects short- and long-term morbidity, mortality, functional recovery, and oncological outcomes [4]. To mitigate these risks, diverting ileostomy (DI) is frequently performed in patients undergoing low anterior resection (LAR) or ultra-low anterior resection (uLAR) to divert the fecal stream and protect the anastomosis. Although a protective stoma may not prevent AL, it may lessen the clinical severity of leakage and decrease the need for urgent reoperation [5].

Nevertheless, ileostomy formation is not without consequences; stoma-related morbidity occurs in approximately 17–45% of patients and may include stoma dysfunction, peristomal dermatitis, electrolyte disturbances, and reductions in quality of life [6,7]. Ileostomy closure (IC) requires a second operative intervention, which carries additional risks, and a substantial proportion of patients never undergo reversal, leading to a permanent stoma despite curative intent [8]. Accordingly, the balance between the potential benefits of fecal diversion and stoma-related morbidity remains an area of ongoing debate. Furthermore, because many stoma-related complications are time-dependent, strategies such as early IC have gained increasing attention as a means of reducing stoma-related morbidity in carefully selected patients [9]. Although the protective role of DI against the clinical consequences of AL has been extensively investigated, additional data are needed to better characterize short-term postoperative morbidity and stoma-related complications in patients undergoing laparoscopic TME after nCRT.

Therefore, this study aimed to evaluate the association between DI and short-term postoperative outcomes, complication severity, readmission, reintervention, and stoma-related morbidity in patients with locally advanced mid- and distal rectal cancer undergoing laparoscopic TME following nCRT.

## 2. Materials and Methods

### 2.1. Study Design and Patient Selection

This retrospective single-center cohort study included patients who underwent surgery for rectal cancer at the Department of General Surgery, Ondokuz Mayıs University Faculty of Medicine, between 1 March 2020 and 1 March 2025. Patient selection was performed according to predefined inclusion and exclusion criteria. Locally advanced rectal cancer was defined as cT3–4 and/or node-positive disease on pretreatment pelvic magnetic resonance imaging (MRI). Patients were eligible for inclusion if they were aged ≥18 years, had histologically confirmed locally advanced mid- or distal rectal adenocarcinoma, received nCRT before TME, underwent elective TME with colorectal or coloanal anastomosis using an initial laparoscopic approach, and had complete demographic, clinical, operative, pathological, and follow-up data available. Patients were excluded if they underwent abdominoperineal resection or planned open surgery; did not receive nCRT; had previous colorectal surgery, synchronous colorectal malignancy, distant metastatic disease, inflammatory bowel disease, or a pre-existing stoma; underwent cytoreductive surgery with hyperthermic intraperitoneal chemotherapy (CRS-HIPEC) or concomitant surgery for another primary malignancy during the same operative session; or had incomplete clinical data. The patient selection process is illustrated in Figure 1. All eligible patients during the study period were included, and the sample size was determined by the number of available cases.

### 2.2. Data Collection and Variables

Clinical, operative, pathological, and follow-up data were retrospectively collected from the institutional electronic medical records. Baseline variables included demographic characteristics, comorbidities, tumor characteristics, neoadjuvant treatment, and operative details. Pathological variables included ypT stage, ypN stage, circumferential resection margin (CRM) status, proximal and distal resection margins, pathological complete response (pCR), and TME specimen quality. TME specimen quality was assessed according to the Quirke classification. Because of the small number of near-complete and incomplete specimens, these categories were combined into a single non-complete category for analysis. The primary outcome was any 30-day postoperative complication after the index operation, intentionally including stoma-related complications to reflect the overall treatment burden associated with DI. Secondary outcomes included complication severity, 30-day readmission, 30-day surgical reintervention, length of hospital stay, 30-day mortality, AL, and ileostomy-related outcomes. AL was defined as disruption of anastomotic integrity detected clinically and/or radiologically and was graded according to the International Study Group of Rectal Cancer (ISREC) classification (grades A–C) [10]. High-output stoma (HOS) was defined as stoma output exceeding 1500 mL/day [11]. Clinically relevant HOS-related dehydration was recorded when medical treatment and/or intravenous fluid replacement was required. For the primary outcome analysis, all postoperative complications occurring within 30 days after the index operation were recorded and graded according to the Clavien–Dindo (CD) classification [12]. Thirty-day readmission, 30-day surgical reintervention, and 30-day mortality were also assessed during this period. In addition, individual postoperative complications, including AL, bleeding, ileus, and urinary complications, were recorded throughout the available follow-up period. Postoperative ileus (POI) was defined as the occurrence of at least two gastrointestinal symptoms that started on postoperative day four or later, including nausea or vomiting, inability to tolerate oral intake for more than 24 h, absence of flatus or bowel movement for over 24 h, abdominal distension, or radiologic findings consistent with ileus [13].

Ileostomy-related outcomes were evaluated separately as formation-related and closure-related complications. Formation-related complications occurring from ileostomy creation until IC or the last follow-up included HOS-related dehydration, peristomal skin complications, ileostomy ischemia, stenosis, prolapse, and stoma retraction. These complications were categorized according to timing as occurring within the first 30 postoperative days or after 30 days until IC or the last follow-up, and cumulative rates over the entire period were also reported. Closure-related complications, including wound infection, incisional hernia, and AL, were assessed within 30 days after IC.

### 2.3. Preoperative Evaluation and Surgical Management

Following endoscopic and histopathological confirmation of rectal cancer, all patients underwent pelvic MRI, upper abdominal MRI, and thoracic computed tomography (CT) for clinical staging, assessment of resectability, and exclusion of distant metastases. Tumor level was classified according to the distance from the anal verge measured on pretreatment pelvic MRI as distal (≤6 cm) or mid (>6 to ≤11 cm). Patients with locally advanced rectal cancer were reviewed by a multidisciplinary tumor board to determine the optimal treatment strategy. Based on multidisciplinary tumor board recommendations, patients received either conventional long-course nCRT or total neoadjuvant therapy (TNT). Long-course nCRT consisted of pelvic radiotherapy (50.4 Gy delivered in 28 fractions) administered concurrently with oral capecitabine. Patients selected for TNT received long-course chemoradiotherapy followed by consolidation FOLFOX chemotherapy before surgery. Restaging with pelvic MRI, upper abdominal MRI, thoracic CT, and rectoscopy was performed 8–10 weeks after completion of neoadjuvant treatment, and TME was initiated using an initial laparoscopic approach 12–16 weeks after completion of neoadjuvant treatment by one of three dedicated colorectal surgeons. Whenever a DI was anticipated, patients were evaluated preoperatively by a dedicated stoma therapist, and the optimal stoma site was routinely marked before surgery. All diverting ileostomies were constructed as loop ileostomies, without the use of stoma rods or bridges. A formal Enhanced Recovery After Surgery (ERAS) pathway was not used during the study period; routine postoperative management, including fluid management, remained consistent throughout the study period. Patients undergoing DI received stoma education and postoperative support from a specialized stoma care team throughout the study period.

Anastomotic integrity was routinely assessed intraoperatively using an air-leak test when technically feasible following colorectal or coloanal anastomosis, and a pelvic drain was routinely placed. Indocyanine green (ICG) fluorescence angiography was not routinely used in all patients and was performed when there was concern regarding anastomotic perfusion and when technically feasible. The decision to construct a DI was based on institutional criteria and intraoperative assessment of the risk of anastomotic failure. According to our institutional practice, factors considered for DI formation included a very low colorectal or coloanal anastomosis, restricted pelvic working space with inadequate visualization and technically demanding pelvic dissection, tissue characteristics following neoadjuvant treatment, impaired anastomotic perfusion on ICG fluorescence angiography when ICG was used, and a positive air-leak test. These factors were assessed collectively by the operating surgeon when determining the need for diversion. Prior to IC, anastomotic healing was confirmed endoscopically and, when indicated, by contrast-enhanced abdominopelvic CT with rectal contrast. In patients with an uncomplicated postoperative course, stoma closure was scheduled at the earliest feasible time following the index operation. IC was performed under general anesthesia through a peristomal approach. Following mobilization of the ileostomy, intestinal continuity was restored using a stapled side-to-side anastomosis. The fascial defect was closed, and the skin was approximated using a purse-string technique.

### 2.4. Statistical Analysis

All statistical analyses were performed using IBM SPSS Statistics, version 27.0 (IBM Corp., Armonk, NY, USA). Continuous variables were assessed for distributional characteristics using the Kolmogorov–Smirnov test. Normally distributed variables were summarized as means ± standard deviations (SDs) and compared using the independent-samples t-test, whereas non-normally distributed variables were summarized as medians and interquartile ranges (IQRs) and compared using the Mann–Whitney U test. Categorical variables were reported as frequencies and percentages and compared using Pearson’s chi-square test or Fisher’s exact test, as appropriate. Unweighted between-group comparisons were considered descriptive.

Because the decision to perform DI was non-randomized and based on institutional criteria and intraoperative assessment of anastomotic risk, propensity score overlap weighting (OW) was used as the primary adjusted analysis to address confounding by indication. The propensity score, defined as the probability of receiving DI, was estimated using logistic regression, including the following prespecified covariates: age; sex; ASA class III–IV versus I–II; any comorbidity; tumor distance from the anal verge on pelvic MRI; advanced clinical T stage (cT3c–cT4 versus cT2–cT3b); cN2 versus cN0–1; neoadjuvant treatment (TNT versus standard nCRT); surgical procedure (uLAR/intersphincteric resection [ISR] versus LAR); and conversion to laparotomy. Covariates were selected a priori based on clinical relevance rather than their associations with the outcomes. Operative duration was not included because it was measured during the operative course and could be influenced by stoma formation, making it a potential post-exposure variable rather than a baseline confounder.

For patients who underwent DI, the overlap weight was calculated as 1 minus the propensity score, whereas for patients without DI, the overlap weight was equal to the propensity score. Covariate balance before and after OW was assessed using standardized mean differences (SMDs), with an absolute SMD < 0.10 indicating adequate balance. The effective sample size was also calculated for the weighted sample. The same overlap weights were used to estimate the association between DI and each postoperative outcome. Overlap-weighted risks were estimated separately for the DI and non-DI groups. Adjusted risk differences (RDs; DI minus non-DI) and their 95% confidence intervals (CIs) were estimated using weighted binomial generalized linear models with an identity link. Adjusted risk ratios (RRs; DI divided by non-DI) and 95% CIs were estimated using weighted Poisson models with a log link and robust sandwich variance estimators. Reported *p*-values were derived from robust Wald tests from the risk-difference models. Any complication within 30 days was analyzed as the overall morbidity outcome and included stoma-related complications. To address the potential structural bias arising from complications that can occur only in patients with a stoma, non-stoma-related complications within 30 days were analyzed separately. CD grade III–V complications, 30-day readmission, 30-day surgical reintervention, and AL were analyzed as additional postoperative outcomes. AL was considered exploratory because only seven events occurred.

As a sensitivity analysis for non-stoma-related complications within 30 days, crude and multivariable Firth-penalized logistic regression models were fitted without OW. The adjusted Firth model included the same prespecified covariates used in the propensity score model, and 95% CIs based on profile penalized likelihood were reported. This sensitivity analysis was performed to assess the robustness of the association between DI and non-stoma-related morbidity using a method that reduces small-sample estimation bias. There were no missing data in the analyzed cohort; therefore, no imputation was performed. All tests were two-sided, and *p* < 0.05 was considered statistically significant. No adjustment for multiple comparisons was performed; secondary and exploratory outcomes were interpreted with emphasis on effect estimates and 95% CIs.

## 3. Results

Of 233 patients assessed for eligibility, 129 were excluded, and 104 were included in the final cohort (Figure 1). A total of 50 patients (48.1%) underwent DI, whereas 54 (51.9%) did not. Baseline characteristics are summarized in Table 1. Tumors were located significantly closer to the anal verge in the DI group according to both pelvic MRI and rectoscopy measurements (both *p* = 0.001). Consequently, distal rectal tumors were more frequent in the DI group, whereas mid-rectal tumors predominated in the non-DI group (*p* < 0.001). No statistically significant between-group differences were observed in pathological characteristics.

Operative and postoperative outcomes are summarized in Table 2. uLAR and ISR were performed significantly more frequently in the DI group, whereas LAR was more common in the non-DI group (*p* < 0.001). Operative durations were longer in the DI group (*p* < 0.001). No statistically significant difference in AL rates was observed between the groups (6.0% vs. 7.4%, *p* > 0.99). However, any 30-day postoperative complication (68.0% vs. 46.3%, *p* = 0.026), severe complications (CD grade III–V: 34.0% vs. 9.3%, *p* = 0.006), and 30-day readmission (42.0% vs. 13.0%, *p* = 0.001) occurred more frequently in the DI group. Surgical reintervention within 30 days was also more frequent in the DI group, although the difference did not reach statistical significance (20.0% vs. 7.4%, *p* = 0.085). Among the 21 patients readmitted within 30 days in the DI group, the primary causes of readmission were HOS-related dehydration in 9 (42.9%), ileostomy-related complications requiring reoperation in 7 (33.3%), postoperative ileus in 3 (14.3%), and AL in 2 (9.5%). In the non-DI group, the primary causes among the seven readmitted patients were AL in three (42.9%), postoperative ileus in three (42.9%), and a urinary complication in one patient (14.3%). The causes of 30-day readmission are detailed in Supplementary Table S1.

AL occurred in four patients in the non-DI group. Two patients underwent anastomotic takedown with end colostomy, whereas the remaining two were managed with pelvic drainage and secondary DI. In the DI group, AL developed in three patients; two were managed conservatively, whereas one underwent transanal repair of the anastomotic defect. Within the first 30 postoperative days, nine patients (18.0%) in the DI group required reoperation because of ileostomy formation-related complications, including stoma retraction (*n* = 4), stoma ischemia (*n* = 3), and stoma prolapse (*n* = 2). The overall 30-day mortality rate was 2.9% (3/104). Three patients died within 30 days: two patients in the non-DI group died from sepsis following AL, whereas one patient in the DI group died from COVID-19. The COVID-19-related death was considered unrelated to the surgical procedure or postoperative surgical complications. Individual postoperative and ileostomy formation-related complications recorded during the available follow-up period are summarized in Table 3. Among the 50 patients who underwent planned DI at the index operation, 41 (82.0%) subsequently underwent IC, whereas 9 (18.0%) did not undergo IC during follow-up. The median time to IC was 78 days (IQR, 33–231 days). The reasons for non-closure were disease progression (*n* = 3), poor general condition (*n* = 2), patient preference (*n* = 2), death before IC (*n* = 1), and anastomotic stricture following leakage (*n* = 1). Among the 41 patients who underwent IC, wound infection occurred in 3 patients (7.3%), incisional hernia in 1 patient (2.4%), and no AL was detected within 30 days following IC. The median follow-up duration for the overall cohort was 26.6 months (IQR, 13.8–46.1 months).

Propensity score overlap weighting retained all 104 patients, with an effective sample size of 65.5 (31.5 in the DI group and 34.0 in the non-DI group). Before weighting, the maximum absolute SMD across the prespecified covariates was 1.106; after weighting, all absolute SMDs were <0.10, indicating adequate covariate balance. In the overlap-weighted analysis, DI was not significantly associated with non-stoma-related complications within 30 days (46.8% vs. 50.1%; adjusted RR, 0.935; 95% CI, 0.566–1.543; *p* = 0.792) or with overall 30-day morbidity (70.6% vs. 54.3%; adjusted RR, 1.301; 95% CI, 0.893–1.895; *p* = 0.158). In contrast, DI was associated with higher risks of CD grade III–V complications (34.0% vs. 7.3%; adjusted RR, 4.658; 95% CI, 1.477–14.688; *p* = 0.004) and 30-day readmission (41.5% vs. 11.3%; adjusted RR, 3.665; 95% CI, 1.397–9.621; *p* = 0.003). No statistically significant associations were observed for 30-day surgical reintervention or AL; the latter was considered exploratory because only seven events occurred (Table 4). In the Firth penalized logistic regression sensitivity analysis, DI was not significantly associated with non-stoma-related complications within 30 days in either the crude model (odds ratio [OR], 1.34; 95% CI, 0.62–2.90; *p* = 0.460) or the adjusted model (OR, 0.88; 95% CI, 0.33–2.31; *p* = 0.798), consistent with the primary overlap-weighted analysis (Supplementary Table S2).

## 4. Discussion

The present study yielded three principal findings. First, DI was preferentially performed in patients with more distal rectal tumors undergoing technically more demanding procedures, including uLAR and ISR. Second, after adjustment for baseline differences using OW, DI was not significantly associated with either overall 30-day morbidity or non-stoma-related complications, whereas its associations with major morbidity and 30-day readmission persisted. Third, serious stoma-related complications observed in the DI group may have represented an important component of this early postoperative treatment burden, emphasizing that the clinical implications of DI extend beyond anastomotic protection alone.

Reported rates of AL following surgery for mid- and low rectal cancer vary considerably, ranging from approximately 5–10% in some series to more than 20% in others, largely because of differences in anastomotic level, duration of follow-up, and definitions of leakage [10,14,15]. Large cohort studies have reported an overall AL rate of approximately 7%, with a higher risk among patients undergoing neoadjuvant treatment for distal rectal tumors [16]. In the present study, no statistically significant difference in AL rates was observed between the DI and non-DI groups (6.0% vs. 7.4%). Although only seven patients developed AL, differences in its clinical management were observed. In the DI group, two of the three leakages were classified as ISREC Grade B and were managed nonoperatively, whereas all four leakages in the non-DI group were ISREC Grade C, with two patients ultimately requiring anastomotic takedown with end colostomy. This observed pattern may be consistent with the traditional rationale for fecal diversion, namely, to mitigate the clinical consequences of AL when it occurs. Given the limited number of AL events, these observations should be interpreted descriptively rather than as evidence that DI reduces the clinical severity of AL. Previous studies have also reported heterogeneous findings regarding the association between DI and AL incidence, although several have suggested that fecal diversion may facilitate less invasive management when leakage occurs [17,18].

Our findings suggest that stoma-related morbidity represents an important component of the early treatment burden associated with DI. Importantly, nine patients (18.0%) required reoperation within 30 days because of ileostomy formation-related complications. These serious stoma-related events may have contributed to the persistent associations of DI with major morbidity and 30-day readmission observed after OW. Within the first 30 postoperative days, HOS-related dehydration occurred in 22.0% of patients, and the 30-day readmission rate reached 42.0%. Taken together, these findings indicate that stoma-specific adverse events contributed meaningfully to the early postoperative treatment burden observed among patients undergoing DI in our cohort.

This observed stoma-related morbidity is consistent with previous reports describing complications associated with DI. Lee et al. [19] reported HOS, postoperative ileus, and readmission rates of 15.9%, 13.6%, and 16.9%, respectively, following restorative rectal resection with DI. Similarly, Van Butsele et al. [20] observed a 25% readmission rate within an enhanced recovery after surgery pathway, with renal complications, postoperative ileus, AL, and HOS accounting for most readmissions. In a propensity score-matched analysis, Wang et al. [21] also demonstrated comparable AL rates between diverted and non-diverted patients, whereas overall complications, severe complications, and early postoperative small-bowel obstruction were more frequent in the DI group. These previous findings provide context for the stoma-related morbidity observed in our selected cohort. Likewise, the systematic review and meta-analysis by Pisarska et al. [22] concluded that although DI may reduce the clinical consequences of AL, these potential benefits should be balanced against the substantial morbidity associated with stoma formation and the requirement for a second operation for IC.

Beyond early postoperative morbidity, failure to achieve IC represents another important consequence of DI. In the present cohort, IC was achieved in 82.0% of patients, whereas 18.0% had not undergone IC by the last available follow-up. The principal reasons for non-closure were disease progression, poor general condition, patient preference, death before ileostomy closure, and anastomotic stricture following leakage. Our findings are consistent with previous reports showing that approximately 15–30% of intended temporary diverting ileostomies remain unclosed during follow-up [23]. Similarly, the prospective multicenter MISSTO study reported an overall IC rate of 84.0% after a median follow-up of nine months, with approximately 16% of ileostomies remaining unclosed and a median time to IC of 146 days [24]. Collectively, these observations underscore that the consequences of DI extend beyond the index operation, as non-closure of an intended temporary ileostomy remains an important long-term outcome.

Given the substantial burden associated with DI, strategies aimed at reducing stoma-related complications have gained increasing attention. Early IC has been shown to be safe and feasible in carefully selected patients and may reduce stoma-related morbidity without increasing postoperative morbidity or anastomotic complications [25]. In addition, ghost ileostomy has emerged as a selective alternative for patients at low-to-intermediate risk of AL, potentially avoiding unnecessary stoma formation while preserving the option of fecal diversion if clinically significant leakage develops [26]. Although these approaches appear promising, careful patient selection remains essential, and further prospective randomized studies are required before their routine implementation can be recommended.

This study has several limitations. First, its retrospective, single-center design and the non-randomized selection of patients for DI introduce an inherent risk of selection bias and confounding by indication. Patients undergoing DI had more distal tumors and more frequently underwent technically demanding procedures, reflecting systematic differences in baseline surgical risk between the groups. Therefore, the higher morbidity observed in the DI group may reflect not only morbidity directly attributable to the stoma, but also the greater technical complexity of surgery and the higher baseline surgical risk of patients in whom DI was selected. Although OW achieved adequate balance across the measured baseline covariates, residual confounding from unmeasured or incompletely measured factors cannot be excluded. Second, the overall 30-day morbidity outcome included stoma-related complications in order to capture the total early postoperative treatment burden. Because these complications are specific to patients with a stoma, this composite outcome may inherently favor the non-DI group when comparing overall morbidity. To address this limitation, non-stoma-related complications within 30 days were evaluated as a separate outcome in the overlap-weighted analysis, with the findings further supported by Firth penalized logistic regression as a sensitivity analysis. Third, the number of AL events was limited, with only seven cases in the entire cohort, precluding robust conclusions regarding the association between DI and either the incidence or clinical severity of AL. Accordingly, the AL findings should be considered exploratory and interpreted descriptively. Fourth, the relatively modest sample size and limited number of events for some outcomes, including only nine cases of ileostomy non-closure, limited the precision of adjusted estimates and precluded a reliable analysis of predictors of non-closure. Fifth, the study included only patients undergoing an initial laparoscopic approach after nCRT, which may limit the generalizability of the findings to other surgical approaches or treatment strategies. Finally, quality-of-life and patient-reported outcomes were not assessed. This represents an important limitation because the impact of temporary ileostomy on quality of life and patient experience is a key component of its overall treatment burden and cannot be adequately captured by postoperative morbidity and readmission rates alone.

## 5. Conclusions

After adjustment for baseline differences using OW, DI was not significantly associated with overall or non-stoma-related 30-day morbidity, whereas its associations with major morbidity and 30-day readmission persisted. Serious stoma-related complications observed in the DI group may have represented an important component of this early postoperative treatment burden. These findings support careful patient selection for DI, particularly when balancing the potential benefits of fecal diversion against stoma-related morbidity.

## Figures and Tables

**Figure 1:**
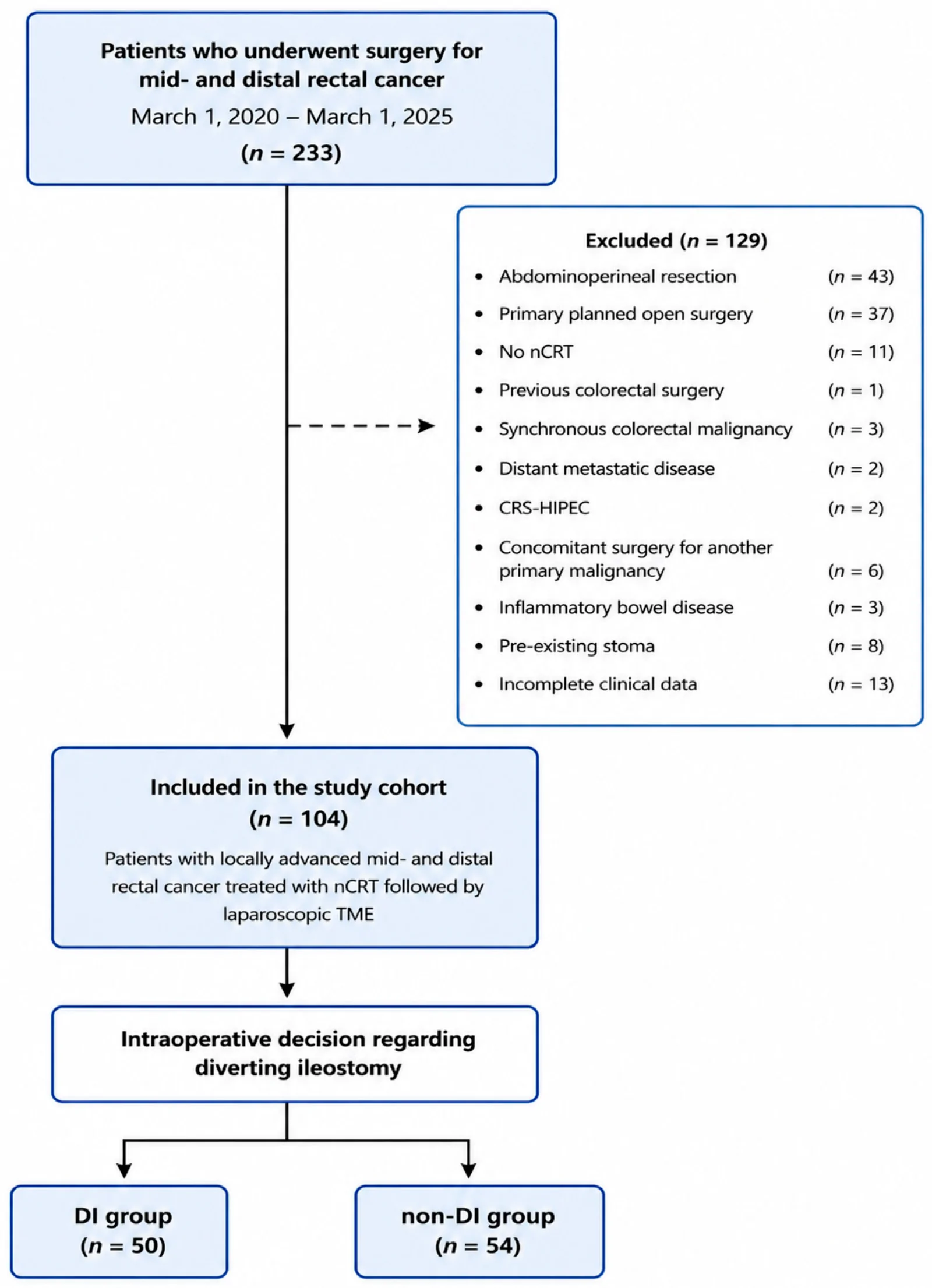
**Flowchart of patient selection for the study cohort.** APR, abdominoperineal resection; CRS-HIPEC, cytoreductive surgery with hyperthermic intraperitoneal chemotherapy; nCRT, neoadjuvant chemoradiotherapy; TME, total mesorectal excision; DI, diverting ileostomy. No missing data in the analyzed cohort; patients with incomplete clinical data were excluded.

**Table 1:** Demographic, clinical, and pathological characteristics of the study population.

Characteristic	DI (*n* = 50)	Non-DI (*n* = 54)	*p*-Value
Sex			0.836
Male	34 (68.0)	35 (64.8)	
Female	16 (32.0)	19 (35.2)	
Age, years, median (IQR)	62.0 (55.5–70.0)	60.0 (51.5–66.8)	0.227
ASA			0.561
I	21 (42.0)	26 (48.1)	
II	6 (12.0)	10 (18.5)	
III	21 (42.0)	16 (29.6)	
IV	2 (4.0)	2 (3.7)	
Comorbidity	24 (48.0)	26 (48.1)	>0.99
Distance from anal verge on pelvic MRI, cm, median (IQR)	5.35 (3.00–7.63)	8.75 (7.00–10.00)	0.001
Distance from anal verge on rectoscopy, cm, median (IQR)	4.00 (2.75–7.00)	9.00 (6.00–9.00)	0.001
Tumor location			<0.001
Mid rectum	22 (44.0)	47 (87.0)	
Distal rectum	28 (56.0)	7 (13.0)	
cT stage			0.708
T2	3 (6.0)	2 (3.7)	
T3a	12 (24.0)	10 (18.5)	
T3b	22 (44.0)	19 (35.2)	
T3c	6 (12.0)	10 (18.5)	
T3d	4 (8.0)	7 (13.0)	
T4a	3 (6.0)	5 (9.3)	
T4b	0	1 (1.9)	
cN stage			0.627
N0	5 (10.0)	5 (9.3)	
N1a	4 (8.0)	1 (1.9)	
N1b	5 (10.0)	9 (16.7)	
N1c	4 (8.0)	3 (5.6)	
N2a	8 (16.0)	7 (13.0)	
N2b	24 (48.0)	29 (53.7)	
Neoadjuvant therapy			0.079
Total neoadjuvant therapy	18 (36.0)	29 (53.7)	
Standard neoadjuvant chemoradiotherapy	32 (64.0)	25 (46.3)	
ypT stage			0.326
T0	12 (24.0)	9 (16.7)	
Tis	1 (2.0)	2 (3.7)	
T1	3 (6.0)	2 (3.7)	
T2	13 (26.0)	7 (13.0)	
T3	20 (40.0)	33 (61.1)	
T4	1 (2.0)	1 (1.9)	
ypN stage			0.225
N0	36 (72.0)	42 (77.8)	
N1a	4 (8.0)	8 (14.8)	
N1b	5 (10.0)	2 (3.7)	
N1c	0	1 (1.9)	
N2a	3 (6.0)	1 (1.9)	
N2b	2 (4.0)	0	
Circumferential resection margin			0.229
Negative	48 (96.0)	54 (100)	
Positive	2 (4.0)	0	
Distal resection margin			0.481
Negative	49 (98.0)	54 (100)	
Positive	1 (2.0)	0	
Pathologic response			0.343
Non-pCR	39 (78.0)	46 (85.2)	
pCR	11 (22.0)	8 (14.8)	
TME quality			0.670
Complete	47 (94.0)	52 (96.3)	
Non-complete	3 (6.0)	2 (3.7)	

**ASA**, American Society of Anesthesiologists; **DI**, diverting ileostomy; **IQR**, interquartile range; **MRI**, magnetic resonance imaging; **pCR**, pathological complete response; **TME**, total mesorectal excision.

**Table 2:** Operative details and postoperative outcomes of the study population.

Characteristic	DI (*n* = 50)	Non-DI (*n* = 54)	*p*-Value
Surgical procedure			<0.001
Low anterior resection	10 (20.0)	33 (61.1)	
Ultra-low anterior resection	19 (38.0)	15 (27.8)	
Intersphincteric resection	21 (42.0)	6 (11.1)	
Operative duration (min), mean ± SD	211 ± 17	171 ± 11	<0.001
Conversion to laparotomy, *n* (%)	10 (20.0)	5 (9.3)	0.164
Anastomotic leakage, *n* (%)	3 (6.0)	4 (7.4)	>0.99
ISREC Grade B	2 (4.0)	0	
ISREC Grade C	1 (2.0)	4 (7.4)	
Complications within 30 days, *n* (%)	34 (68.0)	25 (46.3)	0.026
Readmission within 30 days, *n* (%)	21 (42.0)	7 (13.0)	0.001
Surgical reintervention within 30 days, *n* (%)	10 (20.0)	4 (7.4)	0.085
Clavien–Dindo classification within 30 days, *n* (%)			0.006
None	16 (32.0)	29 (53.7)	
Mild (I–II)	17 (34.0)	20 (37.0)	
Severe (III–V)	17 (34.0)	5 (9.3)	
30-day mortality, *n* (%)	1 (2.0)	2 (3.7)	>0.99
Length of hospital stay (days), median (IQR)	7.0 (6.0–9.0)	8.0 (7.0–9.8)	0.235

**DI**, diverting ileostomy; **IQR**, interquartile range; **ISREC**, International Study Group of Rectal Cancer; **SD**, standard deviation. No Grade A anastomotic leakage occurred in either group.

**Table 3:** Individual postoperative complications and timing of ileostomy formation-related complications.

Complications, *n* (%)	DI (*n* = 50)	Non-DI (*n* = 54)	*p*-Value
Postoperative complications during the available follow-up period			
Bleeding	4 (8.0)	3 (5.6)	0.708
Anastomotic leakage	3 (6.0)	4 (7.4)	>0.99
Ileus	14 (28.0)	7 (13.0)	0.086
Urinary complications	5 (10.0)	8 (14.8)	0.559
Ileostomy formation-related complications	**Within 30 days, *n* (%)**	**>30 days until IC/last follow-up, *n* (%)**	**Cumulative until IC/last follow-up, *n* (%)**
HOS-related dehydration	11 (22.0)	2 (4.0)	13 (26.0)
Peristomal skin complications	10 (20.0)	2 (4.0)	12 (24.0)
Retraction	4 (8.0)	—	4 (8.0)
Ischemia	3 (6.0)	2 (4.0)	5 (10.0)
Prolapse	2 (4.0)	—	2 (4.0)
Stenosis	—	1 (2.0)	1 (2.0)

**DI**, diverting ileostomy; **HOS**, high-output stoma; **IC**, ileostomy closure. Postoperative complications were recorded during the available follow-up period. Ileostomy formation-related complications are presented separately according to timing: within the first 30 postoperative days, after 30 days until IC or the last available follow-up, and cumulatively until IC or the last available follow-up. For patients whose ileostomy was not closed, the last available follow-up was used as the endpoint. Ileostomy formation-related complications were not mutually exclusive; individual patients could experience more than one complication.

**Table 4:** Propensity score overlap-weighted associations between diverting ileostomy and postoperative outcomes.

Outcome	DI Risk, %	Non-DI Risk, %	Adjusted Risk Difference, Percentage Points (95% CI)	Adjusted Risk Ratio (95% CI)	*p*-Value
Non-stoma-related complications within 30 days	46.8	50.1	−3.3 (−27.5 to 21.0)	0.935 (0.566–1.543)	0.792
Any complication within 30 days	70.6	54.3	16.3 (−6.3 to 39.0)	1.301 (0.893–1.895)	0.158
Clavien–Dindo grade III–V complications	34.0	7.3	26.7 (8.7 to 44.7)	4.658 (1.477–14.688)	0.004
Readmission within 30 days	41.5	11.3	30.1 (10.4 to 49.9)	3.665 (1.397–9.621)	0.003
Surgical reintervention within 30 days	19.6	6.0	13.6 (−1.7 to 28.8)	3.251 (0.818–12.923)	0.081
Anastomotic leakage	9.1	6.0	3.1 (−10.0 to 16.3)	1.518 (0.277–8.298)	0.642

**CI**, confidence interval; **DI**, diverting ileostomy. Risk differences are expressed in percentage points. *p*-values were derived from robust Wald tests from the risk-difference models. Anastomotic leakage was considered exploratory because only seven events occurred.

## Data Availability

The data analyzed are available from the corresponding author upon reasonable request.

## Supplementary Materials

The following supporting information can be downloaded at: https://www.mdpi.com/article/10.3390/jcm15197418/s1, Table S1. Primary cause of 30-day readmission. Table S2. Firth penalized logistic regression analysis for non-stoma-related complications within 30 days.

## References

[1] Kotek J., Cyrany J., Sirový M., Novotný P., Páral J. (2025). Modern approaches to rectal cancer: Integrating endoscopic, surgical, and oncological care. Cancers.

[2] van der Pas M.H.G.M., Haglind E., Cuesta M.A., Fürst A., Lacy A.M., Hop W.C.J., Bonjer H.J., Colorectal Cancer Laparoscopic or Open Resection II (COLOR II) Study Group (2013). Laparoscopic versus open surgery for rectal cancer (COLOR II): Short-term outcomes of a randomised, phase 3 trial. Lancet Oncol..

[3] Jeong S.Y., Park J.W., Nam B.H., Kim S., Kang S.B., Lim S.B., Choi H.S., Kim D.W., Chang H.J., Kim D.Y. (2014). Open versus laparoscopic surgery for mid-rectal or low-rectal cancer after neoadjuvant chemoradiotherapy (COREAN trial): Survival outcomes of an open-label, non-inferiority, randomised controlled trial. Lancet Oncol..

[4] Cong Z.J., Hu L.H., Xing J.J., Bian Z.Q., Fu C.G., Yu E.D., Li Z.S., Zhong M. (2014). Incidence and mortality of anastomotic dehiscence requiring reoperation after rectal carcinoma resection. Int. Surg..

[5] Matthiessen P., Hallböök O., Rutegård J., Simert G., Sjödahl R. (2007). Defunctioning stoma reduces symptomatic anastomotic leakage after low anterior resection of the rectum for cancer: A randomized multicenter trial. Ann. Surg..

[6] Kim M.J., Kim Y.S., Park S.C., Sohn D.K., Kim D.Y., Chang H.J., Oh J.H. (2016). Risk factors for permanent stoma after rectal cancer surgery with temporary ileostomy. Surgery.

[7] Man V.C.M., Choi H.K., Law W.L., Foo D.C.C. (2016). Morbidities after closure of ileostomy: Analysis of risk factors. Int. J. Colorectal Dis..

[8] Snijders H.S., van Leersum N.J., Henneman D., de Vries A.C., Tollenaar R.A.E.M., Stiggelbout A.M., Wouters M.W.J.M., Dekker J.W.T. (2015). Optimal treatment strategy in rectal cancer surgery: Should we be cowboys or chickens?. Ann. Surg. Oncol..

[9] Gouvas N., Manatakis D., Agalianos C., Dimitriou N., Baloyiannis I., Tzovaras G., Xynos E. (2024). Defunctioning ileostomy after low anterior resection of rectum: Morbidity related to fashioning and closure. Medicina.

[10] Rahbari N.N., Weitz J., Hohenberger W., Heald R.J., Moran B., Ulrich A., Holm T., Wong W.D., Tiret E., Moriya Y. (2010). Definition and grading of anastomotic leakage following anterior resection of the rectum: A proposal by the International Study Group of Rectal Cancer. Surgery.

[11] Babakhanlou R., Larkin K., Hita A.G., Stroh J., Yeung S.C. (2022). Stoma-related complications and emergencies. Int. J. Emerg. Med..

[12] Dindo D., Demartines N., Clavien P.A. (2004). Classification of surgical complications: A new proposal with evaluation in a cohort of 6336 patients and results of a survey. Ann. Surg..

[13] Goldenshluger M., Gruper O., Anderson Y., Caller T., Katz E., Lebedeyev A., Kent I., Ram E., Lutrin D., Segev L. (2026). Postoperative ileus after rectal cancer surgery: Assessing incidence, severity, and impact across open, laparoscopic, and robotic approaches. J. Clin. Med..

[14] Litchinko A., Buchs N., Balaphas A., Toso C., Liot E., Meurette G., Ris F., Meyer J. (2024). Score prediction of anastomotic leak in colorectal surgery: A systematic review. Surg. Endosc..

[15] Serra-Aracil X., Zarate A., Bargalló J., Gonzalez A., Serracant A., Roura J., Delgado S., Mora-López L., Ta-LaTME Study Group (2023). Transanal versus laparoscopic total mesorectal excision for mid and low rectal cancer (Ta-LaTME study): Multicentre, randomized, open-label trial. Br. J. Surg..

[16] Marino M., Verberne C., Merchant J.F., Hassan S.A., Witjes C.D.M., Lindsey I. (2025). Balancing risk in rectal cancer surgery: A retrospective cohort study examining the consequences of anastomotic leaks and diversion. Turk. J. Colorectal Dis..

[17] Munshi E., Lydrup M.L., Buchwald P. (2023). Defunctioning stoma in anterior resection for rectal cancer does not impact anastomotic leakage: A national population-based cohort study. BMC Surg..

[18] Hrebinko K., Anto V.P., Reitz K.M., Gamboa A.C., Regenbogen S.E., Hawkins A.T., Hopkins M.B., Ejaz A., Bauer P.S., Wise P.E. (2024). Prophylactic defunctioning stomas improve clinical outcomes of anastomotic leak following rectal cancer resections: An analysis of the US Rectal Cancer Consortium. Int. J. Colorectal Dis..

[19] Lee N., Lee S.Y., Kim C.H., Kwak H.D., Ju J.K., Kim H.R. (2021). The relationship between high-output stomas, postoperative ileus, and readmission after rectal cancer surgery with diverting ileostomy. Ann. Coloproctol..

[20] van Butsele J., Bislenghi G., D’Hoore A., Wolthuis A.M. (2021). Readmission after rectal resection in the ERAS-era: Is a loop ileostomy the Achilles heel?. BMC Surg..

[21] Wang Y., Wang X., Huang S., Zhu H., Huang Y. (2024). Impact of diversion ileostomy on postoperative complications and recovery in the treatment of locally advanced upper-half rectal cancer. Sci. Rep..

[22] Pisarska M., Gajewska N., Małczak P., Wysocki M., Witowski J., Torbicz G., Major P., Mizera M., Dembiński M., Migaczewski M. (2018). Defunctioning ileostomy reduces leakage rate in rectal cancer surgery: Systematic review and meta-analysis. Oncotarget.

[23] Civil O., Sakoglu N., Tekin A., Kement M. (2026). Risk factors for non-reversal of diverting ileostomies: A retrospective analysis of 456 patients. Int. J. Colorectal Dis..

[24] Ferrara F., Rizzo G., Bondurri A., Forni C., Anania G., Anastasi A., Baiocchi G.L., Boccia L., Cassini D., Catarci M. (2025). Outcomes of loop ileostomy after rectal resection for cancer: A prospective observational multicenter snapshot study from Multidisciplinary Italian Study group for STOmas (MISSTO). Tech. Coloproctol..

[25] Podda M., Coccolini F., Gerardi C., Castellini G., Wilson M.S.J., Sartelli M., Pacella D., Catena F., Peltrini R., Bracale U. (2022). Early versus delayed defunctioning ileostomy closure after low anterior resection for rectal cancer: A meta-analysis and trial sequential analysis of safety and functional outcomes. Int. J. Colorectal Dis..

[26] McKechnie T., Lee J., Lee Y., Tessier L., Amin N., Doumouras A., Hong D., Eskicioglu C. (2023). Ghost ileostomy versus loop ileostomy following oncologic resection for rectal cancer: A systematic review and meta-analysis. Surg. Innov..

