# Supplementary material for: Short-Term Outcomes and Stoma-Related Morbidity of Diverting Ileostomy After Laparoscopic Total Mesorectal Excision Following Neoadjuvant Chemoradiotherapy for Mid- and Distal Rectal Cancer: A Retrospective Single-Center Study
Source: J Clin Med. 2026 Sep 24;15(19):7418. doi: 10.3390/jcm15197418 (PMC13666439; doi:10.3390/jcm15197418)
Supplement: Supplementary file 1 [file jcm-15-07418-s001.zip › jcm-4518626-supplementary.pdf]

Supplementary Table S1. Primary cause of 30-day readmission.

Primary cause of 30-day readmission	DI (n=21)	Non-DI (n=7)
HOS-related dehydration	9 (42.9%)	0
Ileostomy-related complication requiring reoperation	7 (33.3%)	0
Postoperative ileus	3 (14.3%)	3 (42.9%)
Anastomotic leakage	2 (9.5%)	3 (42.9%)
Urinary complication	0	1 (14.3%)
**Total**	**21 (100%)**	**7 (100%)**

DI, diverting ileostomy; **HOS**, high-output stoma. Percentages were calculated based on the number of readmitted patients in each group.

Supplementary Table S2. Firth penalized logistic regression analysis for non-stoma-related complications within 30 days.

Model	OR	95% CI	p value
Crude model	1.34	0.62–2.90	0.460
Adjusted model	0.88	0.33–2.31	0.798

The outcome was non-stoma-related complications within 30 days after the index operation. The adjusted Firth-penalized logistic regression model included the same prespecified covariates used in the propensity score model: age, sex, ASA class (III–IV vs. I–II), any comorbidity, tumor distance from the anal verge on pelvic MRI, advanced clinical T stage (cT3c–cT4 vs. cT2–cT3b), cN2 vs. cN0–1, neoadjuvant treatment (TNT vs. standard nCRT), surgical procedure (uLAR/ISR vs. LAR), and conversion to laparotomy. ORs are presented with profile-penalized likelihood 95% CIs.
CI, confidence interval; **OR**, odds ratio.
